# Are Differences in Bedtimes Bad for Relationships? Associations With Attachment and Conflict in Middle-Aged Couples

**DOI:** 10.1093/geroni/igaa057.1518

**Published:** 2020-12-16

**Authors:** Joshua Novak, Stephanie Wilson

**Affiliations:** 1 Auburn University, Auburn, Alabama, United States; 2 Southern Methodist University, Dallas, Texas, United States

## Abstract

A robust body of literature has found birdirectional associations between sleep quality and marital quality in couple relationships (Hasler & Troxel, 2010; Pearlin, 2010). Additionally, dyadic research shows that differences in couples’ bed time routines and habits is associated with mental health outcomes (Chen, 2018), however the literature has not connected them with other marital processes that are mutable and clinically relevant. Attachment theory provides a clinically relevant framework that captures both interpersonal marital processes such as relationship conflict as well intrapersonal processes of individual emotional safety—essentially individuals’ personal strategies to balance closeness and distance in a relationship (Feeney, 2002; Rhodes et al., 2001). The two main attachment styles related to sleep processes are attachment avoidance and attachment anxiety (Collins et al., 2002; Gun, 2015; Troxel, 2007). Utilizing data from 234 couple dyads, we investigated if differences in partners’ bed times is associated with conflict frequency and attachment avoidance using a structural equation modeling approach. We controlled for a number of important factors and tested our hypothesized model against two plausible alternative models. Results revealed that greater difference in partners’ bed times was associated with higher conflict frequency for both husbands and wives through higher men’s attachment avoidance. Our findings highlight previous research on matched vs. unmatched couples on sleep routines, habits, and chronotypes (both morning or night vs. different; Larson et al., 1991) but highlight mutable and clinically relevant constructs for intervention. Implications for health promotion and marital therapy will be discussed.

